# Axon and Myelin Morphology in Animal and Human Spinal Cord

**DOI:** 10.3389/fnana.2017.00129

**Published:** 2017-12-22

**Authors:** Ariane Saliani, Blanche Perraud, Tanguy Duval, Nikola Stikov, Serge Rossignol, Julien Cohen-Adad

**Affiliations:** ^1^NeuroPoly Lab, Institute of Biomedical Engineering, Polytechnique Montreal, Montreal, QC, Canada; ^2^Montreal Heart Institute, Montreal, QC, Canada; ^3^Groupe de Recherche sur le Système Nerveux Central, Department of Neuroscience, Faculty of Medicine, Université de Montréal, Montreal, QC, Canada; ^4^Functionnal Neuroimaging Unit, Centre de Recherche de l'Institut Universitaire de Gériatrie de Montréal, Université de Montréal, Montreal, QC, Canada

**Keywords:** spinal cord, white matter, axons, myelin, histology, morphology

## Abstract

Characterizing precisely the microstructure of axons, their density, size and myelination is of interest for the neuroscientific community, for example to help maximize the outcome of studies on white matter (WM) pathologies of the spinal cord (SC). The existence of a comprehensive and structured database of axonal measurements in healthy and disease models could help the validation of results obtained by different researchers. The purpose of this article is to provide such a database of healthy SC WM, to discuss the potential sources of variability and to suggest avenues for robust and accurate quantification of axon morphometry based on novel acquisition and processing techniques. The article is organized in three sections. The first section reviews morphometric results across species according to range of densities and counts of myelinated axons, axon diameter and myelin thickness, and characteristics of unmyelinated axons in different regions. The second section discusses the sources of variability across studies, such as age, sex, spinal pathways, spinal levels, statistical power and terminology in regard to tracts and protocols. The third section presents new techniques and perspectives that could benefit histology studies. For example, coherent anti-stokes Raman spectroscopy (CARS) imaging can provide sub-micrometric resolution without the need for fixation and staining, while slide scanners and stitching algorithms can provide full cross-sectional area of SC. In combination with these acquisition techniques, automatic segmentation algorithms for delineating axons and myelin sheath can help provide large-scale statistics on axon morphometry.

## Introduction

Pathologies affecting the spinal cord (SC) could have diverse origins, from vascular diseases, cancer and trauma to neurodegenerative diseases such as multiple sclerosis. In many instances, there is limited knowledge about the actual pathophysiology of the disease, about the succession of neurotoxic events following a trauma (secondary injuries, Tator and Fehlings, 1991; Park et al., 2004), or even the precise vascularization depending on the tract. These issues make it difficult to assess accurately the extent of spinal damage.

SC research is commonly conducted in animal models to improve our understanding of a given pathology, its etiology and evolution, and to develop new therapies (Nout et al., 2012). Mice and rats, despite differences in the structural organization of the SC compared to humans, are one of the most commonly used animals for studying the SC due to the low cost, easy access, and well-established anatomical and functional analysis techniques (Nout et al., 2012; Lee and Lee, 2013; Silva et al., 2014). Cats have also been quite popular in electrophysiological and physiological studies of the SC since they have similar neuroanatomical features to those in humans with regard to SC architecture (Rossignol, 2006). In addition, the capacity of cats to regain locomotion after SCI makes them good candidates for SCI and remyelination studies (Rossignol, 2006; Lee and Lee, 2013; Silva et al., 2014). Lastly, monkeys are another model commonly used to deal with the significant differences in neuroanatomy existing between rats and primates (including humans). Some non-human primates are particularly interesting because of their bipedal locomotion (Nout et al., 2012). When possible, human tissue can be used for anatomical investigation, although several problems arise with the lack of perfusion and subsequent degradation of myelin sheaths in the SC. To maximize the outcome of these studies, it is essential to have access to normative values of axon morphometry (e.g., shape, density, degree of myelination) across various tracts and species. The existence of an exhaustive database recapitulating decades of work on the SC WM by experts in the field would be helpful in that regard.

In addition, novel non-invasive biomarkers based on quantitative magnetic resonance imaging have shown great potential for characterizing axonal damage, which is not easily detectable with conventional MRI. Although these advanced biomarkers are still limited by the spatial resolution MRI, they can provide average quantitative values of axon features within an MR voxel (~1 mm^3^), such as axon diameter distribution (Assaf et al., 2008; Barazany et al., 2009; Huang et al., 2015) and myelin density (Laule et al., 2006; Stikov et al., 2011, 2015; Chen et al., 2013; Fjær et al., 2013; Duval et al., 2015). Applicable in the future for clinical diagnosis and drug development, these biomarkers are still under development and their use requires further validation, notably through comparison with histological data. The existence of an exhaustive database of axon microstructure would thus facilitate the validation of MRI metrics.

While many papers have been published on the characterization of WM axons in the SC, there is a need to summarize systematically those findings, compare them and discuss their possible limitations. The goals of this review article are therefore: (i) to systematize the existing knowledge about SC WM microstructure in different animal species, (ii) to discuss the potential sources of variability associated with these reports and (iii) to suggest optimal protocols and present novel strategies for large-scale histology of the SC. The functional aspects regarding the tracts are briefly mentioned in the conclusion.

## Morphometry of spinal cord microstructure

**Figure 1:**
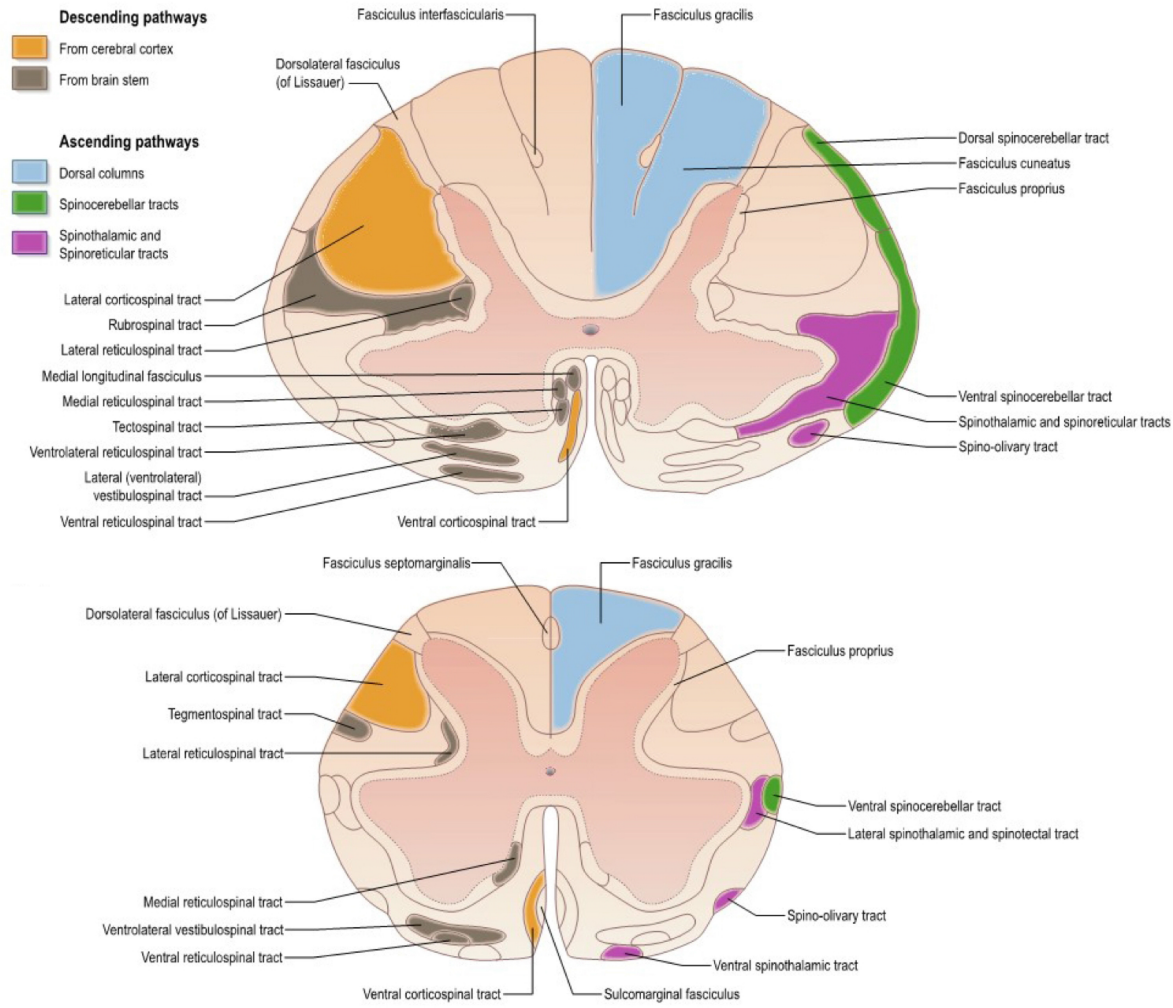
Atlas representing the location of white matter tracts in the human spinal cord at **(Top)** mid-cervical and **(Bottom)** lumbar levels. Reproduced with permission from Standring (2008).

The SC is composed of several tracts (represented for humans in Figure 1), each with its particular role in the nervous system. Descending tracts [lateral motor system: corticospinal (CST) and rubrospinal tracts; medial motor system: reticulospinal and vestibulospinal tracts] are involved in motor control whereas ascending tracts (dorsal columns including fasciculus gracilis and cuneatus, spinothalamic, spinocerebellar, spinoreticular tracts) transmit sensory information including proprioception (Lawrence and Kuypers, 1968; Standring, 2008). Because all those tracts can be affected by pathology, it is important to characterize them individually.

As shown later on, the pyramidal tract has been the most studied one. Although the distinction between this tract and the corticospinal one is of particular importance (considering the termination of corticobulbar fibers in the brainstem) (Leenen et al., 1985; Brodal, 2010), terminology is sometimes imprecise thereby creating some confusion in the reporting (e.g., studies reporting microstructural information on the pyramidal tract at the cervical level).

### Range of densities and counts of myelinated axons in different tracts

Table 1 reports densities and counts of myelinated fibers from various species (rats, cats, monkeys, humans). One point of uncertainty when creating the following table arose from the terminology used in some articles. Sometimes, when employing the word “fibers,” authors did not explicitly mention if they were referring to myelinated fibers only or to all fibers (myelinated and unmyelinated). When applicable this distinction was indicated.

**Table 1:** Counts and densities of myelinated fibers in nervous tissue (brainstem and spinal cord) in various species.

**Species**	**Anatomical location**	**Density of myelinated fibers per mm^2^**	**Number of myelinated fibers**	**Microscopy**	**References**
Rat	Pyramidal tract (brainstem)		91,000	Electron	Leenen et al., 1982
	Pyramidal tract (brainstem)	378,300	140,000	Electron	Leenen et al., 1985
	Pyramidal tract (spinal cervical 2 level)^a^	483,300	43,000	Electron	Leenen et al., 1985
	Sacral spinal cord ; Lateral funiculus		55,000	Electron	Chung and Coggeshall, 1983
	Sacral spinal cord; ventral funiculus		26,000	Electron	Chung and Coggeshall, 1983
	Pyramidal tract (brainstem)		88,908–144,704 (mean 111,600)^a^	Both	Dunkerley and Duncan, 1969
	Pyramidal tract		73,000	Probably optical	Lassek and Rasmussen, 1940
	Pyramidal tract (cervical)		137,000^a^	Optical	Brown, 1971
	Pyramidal tract (brainstem)		188,395–163,530	Electron	Harding and Towe, 1985
Cat	Pyramidal tract (brainstem)		186,000^a^		Lassek and Rasmussen, 1940
	Pyramidal tract (brainstem)		80,000^a^ (great variations: 56,000–106,000)	Optical	van Crevel and Verhaart, 1963
	Pyramidal tract (brainstem)		368,044	Electron	Thomas et al., 1984
	Dorsal funiculus, sacral 2 spinal level		34,520 ± 2,436	Electron	Chung et al., 1985
	Lateral funiculus, sacral 2 spinal level		159,774 ± 7,762	Electron	Chung et al., 1985
	Ventral funiculus, sacral 2 spinal level		74,431 ± 17,936	Electron	Chung et al., 1985
	Pyramidal tract (brainstem)	143,291	356,583	Electron	Biedenbach et al., 1986
Monkey	Pyramidal tract		554,000^b^	Optical	Lassek and Rasmussen, 1940
	Pyramidal tract		435,627^b^	Optical	Russel and Demyer, 1961
	Pyramidal tract		594,000	Electron	Firmin et al., 2014
Human	Pyramidal tract		1,087,200^c^		Demyer, 1959
	Pyramidal tract	101,400^b^			Towe, 1973
	Pyramidal tract (brainstem)	50,000–100,000	663,000–884,000	Electron	Graf and Schramm, 1983
	Pyramidal tract	8,400–9,210	48,768–67,419^c^	Optical	Wada et al., 2001
	Fasciculus gracilis (C3)	19,647–35,773 (mean: 25,267)			Ohnishi et al., 1976
	Fasciculus gracilis (T5)	17,804–29,486 (mean: 23,069)			Ohnishi et al., 1976

a *The terminology used is the authors'. Strictly speaking, the pyramidal tract is only in the midbrain and not in the SC*.

b *In some cases, there was uncertainty about whether the authors included only myelinated or both myelinated and unmyelinated fibers in their counts, as mentioned by this note*.

c *In other cases, the authors specified that their counts included unmyelinated fibers*.

One notable observation emerging from this table is the variability in densities and counts from one specimen to another [e.g., density of 152,000 myelinated axons per mm^2^ vs. 125,000 (Biedenbach et al., 1986)], as well as from one study to the next [e.g., for the human pyramidal tract, Demyer (1959) counted 1,000,000 myelinated fibers against 49,000–67,000 for Wada et al. (2001)].

As a general trend, different fiber counts are associated with different spinal levels. For example, the pyramidal tract of the rat in the brainstem presents roughly between 90,000 and 140,000 fibers, depending on the study, whereas the CST at the cervical 2 level seems to be constituted by about 43,000 fibers (Leenen et al., 1985).

#### Inter-species variability in fiber counts

It is proposed that from one species to the next some tracts gain in importance at the expense of others. The CST tract with its direct corticomotor connections is phylogenetically recent (Heffner and Masterton, 1983; Brodal, 2004; Schieber, 2007; Watson et al., 2009; Towe and Luschei, 2013), and is associated with a reduction of the rubrospinal tract in primates and in humans (Nathan and Smith, 1982; Gramsbergen, 2005; Schieber, 2007; Standring, 2008; Zelenin et al., 2010; de Oliveira-Souza, 2012). Indeed, for locomotion or movement, mammals (except humans) tend to employ more their non-corticospinal descending pathways (Schieber, 2007; Standring, 2008). This observation could be a first explanation for the larger number of fibers in the CST in monkeys than in rats (Nathan and Smith, 1982). A second explanation, likely complementary, would be the requirement of additional CST fibers due to the presence of direct corticomotor connections, leading to a larger CST (Uk and Al, 2006; Spence, 2009). Lastly, Heffner and Masterton (1975) pointed out a correlation between the length of the CST tract and the species' dexterity, which would imply longer tracts for primates. This would explain the inter-species variability in lower segments of the SC (e.g., sacral or lumbar levels) (Iwaniuk et al., 1999).

### Axon diameter and myelin thickness across species

Axon diameter has been measured in several species. The numbers are reported in Table 2 along with the myelin sheath thickness. Note that in previously-published studies the word “fiber diameter” sometimes included the surrounding myelin sheath and sometimes not, without clear mention of it.

**Table 2:** Morphometric data on myelinated axons in nervous tissue obtained from various species.

**Species**	**Anatomical location**	**Mean axonal diameter**	**Axonal diameter range**	**Diameter distribution**	***g*-ratio**	**Myelin thickness**	**References**
Rat	Pyramidal tract (brainstem)	0.80 1.19 (fibers)	0.10–4 0.25–5 (fibers)	90% < 1.5 μm			Leenen et al., 1982^b^
	Pyramidal tract (brainstem and cervical 2 spinal level)	0.72 1.08 (fiber)	0.13–4.92 0.25–6.03 (fiber)		0.65	0.2	Leenen et al., 1985^b^
	Pyramidal tract (midbulbar)	1.054 (fiber) 1.182 (fiber, with shrinkage correction)	Max. 3.7 and 5.2 (fibers)	50% < 1.2 μm (fibers)			Harding and Towe, 1985^a^
	Corticospinal tract (cervical 2)	0.68 (ipsilateral ventral) 0.72 (main)					Brösamle and Schwab, 2000^a^
	Corticospinal tract (cervical)		0.5–3				Joosten and Gribnau, 1988^a^
Cat	Pyramidal tract (brainstem)		<10 (fibers)	73% 0–2 μm 20% 2–4 μm 5% 4–6 μm 2% > 6 μm (fibers)			van Crevel and Verhaart, 1963^b^
	Pyramidal tract (brainstem)	1.98 (median: 1.60)	0.25–23	> 50% < 2 μm 1% > 9 μm 25% 0.5–1 μm 20% 1–1.5 μm			Biedenbach et al., 1986^a^
	Fasciculus gracilis (cervical)		<1–15	97% < 8 μm 50% 2–5 μm			Thomas et al., 1984^a^
Monkey	Pyramidal tract (brainstem)		0.4–6				Ralston et al., 1987^a^
	Pyramidal tract (brainstem)	0.91 (median: 0.68) Fiber: 1.32 (median: 0.97)	0.04–9.48	52% < 1 μm 14% < 0.5 μm			Firmin et al., 2014^a^
Human	Pyramidal tract (brainstem)			70% < 1 μm 84% < 2 μm (fibers)			Lassek and Rasmussen, 1940^b^
	Pyramidal tract (brainstem)		1–20 (fibers)	90% > 1 μm (fibers)			Verhaart, 1947^b^
	Pyramidal tract (brainstem)		~0.3–10	88% < 4 μm 10.77% 4–10 μm 1.4% > 10 μm (fibers)	0.6 for axon < 0.5 μm >0.6 for axon >0.5 μm		Terao et al., 1994^a^

Studies marked with (

a ) have been conducted through electron microscopy, the ones marked with

(

b *) have been conducted with optical microscopy*.

The average diameter in the pyramidal tract of the rat varies between 0.72 μm (Leenen et al., 1985; Brösamle and Schwab, 2000) and 1.182 μm (Harding and Towe, 1985). Variations could be due to different shrinkage (i.e., different protocols), inter-individual differences, or due to whether the myelin sheath was included in the measurement. The distribution of calibers is broad (ranging from 0.1 to 6 μm) but more than 90% of axons are smaller than 1.5 μm. In the corticospinal tract, the average diameter is 0.72 and 0.68 μm (respectively for the main and the ipsilateral ventral components) (Brösamle and Schwab, 2000).

In the cat, the only average axon diameter available is in the pyramidal tract, with a value of 1.98 μm (Biedenbach et al., 1986). In this tract, diameters found were as low as 0.25 μm and up to 23 μm (Biedenbach et al., 1986), but the pyramidal tract is generally composed of thin fibers with the majority of axons smaller than 2 μm (van Crevel and Verhaart, 1963; Biedenbach et al., 1986). Note that a maximal diameter of 23 μm is quite high when comparing to the largest human myelinated fibers [20 μm with myelin sheath in the highest cases (Standring, 2008)], or to the results obtained for the monkey. One has to keep in mind the possibility of histological distortion or the possibility that authors included the myelin sheath in the measurement (this was not specified).

In the monkey, the average diameter in the pyramidal tract is 0.91 μm (Firmin et al., 2014). Axon caliber distribution is broad, ranging from 0.04 to 9.48 μm. There is also a large presence of small fibers, with 52% of myelinated fibers presenting axons smaller than 1 μm (Firmin et al., 2014). In addition, the tract is reported to be heavily myelinated (99% of axons) (Firmin et al., 2014).

Axons of the pyramidal tract in humans were found to be as thin as 0.3 μm (Graf and Schramm, 1983) and as large as 20 μm (Verhaart, 1947), with a majority of thin fibers (about 84%) smaller than 2 μm. This maximal diameter is high, even for human fibers, and possibly explained by inclusion of the myelin sheath. In the lateral CST, predominance of small fibers over large ones was shown to be more pronounced at cervical level 6 and lumbar level 4 than at thoracic level 7 (Terao et al., 1994). In a similar way, according to Ohnishi et al. (1976) in the fasciculus gracilis of humans, axon diameter is smaller at C3 than at T5.

#### Comments on previous reports

All studies seem to have characterized the pyramidal and corticospinal tracts as being mostly thin fibered with a unimodal distribution of axon diameter, regardless of the species. A few large fibers are scattered across the tract. There is a tendency for smaller species to have smaller axons, while larger species present higher maximal axonal diameters (Leenen et al., 1982; Biedenbach et al., 1986). To our knowledge, the spatial distribution of those large axons has not been studied. Rather significant discordance can be observed across studies on the same species when looking at numerical values: a trend in median axonal diameter is difficult to establish.

If larger species have higher maximal diameters, they still present very thin axons thereby providing them with a broader range of axonal diameters. Since the largest fibers of the pyramidal tract represent only a small fraction of the fibers the median diameter does not change very much across species. It is possible that the majority of the “new” fibers (excluding the new larger ones) would be rather small fibers, leading to a stable median axonal diameter. There is therefore an interest in reporting the distribution and the median as opposed to only the mean axonal diameter.

The thickness of the myelin sheath is a parameter of interest in SC morphometry. One way to express it is through the *g*-ratio, which is defined as the ratio between the inner axonal diameter of the fiber to the external diameter or total diameter (including the myelin sheath). Conduction velocity of electrical impulse is maximal with a *g*-ratio of 0.6–0.7 (Rushton, 1951; Leenen et al., 1985; Johansen-Berg and Behrens, 2013). However, this is not a general rule. For instance, large axons seem to have higher g-ratio (Hildebrand and Hahn, 1978; Biedenbach et al., 1986; Johansen-Berg and Behrens, 2013). Furthermore, several studies have reported variations on the degree of myelination of axons presenting the same diameter, in rat and human tissue: for small axons the myelin thickness could vary by up to a factor 4 (Graf and Schramm, 1983; Harding and Towe, 1985).

#### Importance of fiber circularity for axonal diameter measures

The form factor of a fiber (or eccentricity) is the ratio of the smallest to the largest diameter (Leenen et al., 1985). In the pyramidal tract of the rat this factor has been measured by Leenen et al. (1985) as 0.75 and 0.70 for myelinated axons, respectively with and without the myelin sheath. Accordingly, the fibers in the cat's pyramidal tract have been characterized by a small but consistent deviation from the circular shape (Biedenbach et al., 1986). Furthermore, in this case, circularity was decreasing with increasing diameter.

Assuming that axons in the SC are perfectly circular, incorrect cutting will translate into elliptic profiles, and the measure of circularity may prove useful to quantify the extent of the distortion. This objective value of circularity would help the reader to control the degree of the histological bias in the reported morphometric values.

### Characteristics (size, density, counts) of unmyelinated axons

Since the 80's, the presence of a high number of unmyelinated axons in the WM of the SC is often reported, particularly in cats and rats (Langford and Coggeshall, 1981; Leenen et al., 1982, 1985; Chung and Coggeshall, 1983; Chung et al., 1985; Harding and Towe, 1985).

#### Range of diameters

The characteristics of unmyelinated fibers have been summarized for the rat, the cat and the monkey in Table 3.

**Table 3:** Morphometric data of unmyelinated fibers in nervous tissue (brainstem or spinal cord).

**Species**	**Tract**	**Anatomical level**	**Axonal diameter range (μm)**	**Mean axonal diameter (μm)**	**Axonal diameter distribution (μm)**	**References**
Rat	Pyramidal	Medullary pyramid	0.05–1	0.16		Leenen et al., 1982
	Corticospinal	Main component	0.72–1.48	0.72 ± 0.05		Brösamle and Schwab, 2000
	Corticospinal	Ipsilateral ventral		0.68 ± 0.04		Brösamle and Schwab, 2000
Cat	Lissauer	Thoracic and lumbar				Chung et al., 1979
	Pyramidal	Medulla pyramid	0.05–0.6	0.18	0.10–0.30 = 94% 0.01% > 0.5	Thomas et al., 1984
	Pyramidal	Sacral 2	0.1–1			Chung et al., 1985
	Pyramidal	Medullary pyramid	0.05–0.6	0.18		Biedenbach et al., 1986
Monkey	Pyramidal	Medullary pyramid	0.07–2.25			Ralston et al., 1987

*All those studies have been conducted with electron microscopy*.

In rats, the pyramidal tract contains unmyelinated axons with diameters between 0.05 and 1 μm, while the mean are around 0.16 μm (Leenen et al., 1982).

In cats, the same tract presents unmyelinated axons with an average diameter of 0.18 μm, but ranging from 0.05 to 0.6 μm (Thomas et al., 1984). On the other hand, one study reported that the CST at sacral 2 level seems to have slightly larger fibers (0.1–1 μm) (Chung et al., 1985).

In monkeys, fibers are characterized by diameters ranging from 0.07 to 2.25 μm, much larger than in the other species (Ralston et al., 1987).

#### Axonal counts and densities

In a similar fashion to myelinated axons, the counts and densities of unmyelinated fibers have been summarized in Table 4, and similar inter-study variability can be observed. In the cat's pyramidal tract, some authors have found as few as 20,000 fibers against 47,000 for others (Thomas et al., 1984; Biedenbach et al., 1986). In addition, Thomas et al. (1984) also presented great inter-individual variability in their study since their counts ranged from 20,000 to 63,000.

**Table 4:** Counts and densities of unmyelinated fibers in brainstem and spinal cord.

**Species**	**Anatomical location**	**Density of unmyelinated fibers per mm^2^**	**Number of unmyelinated fibers**	**Proportion of unmyelinated fibers (%)**	**References**
Rat	Pyramidal tract (brainstem)	518,700	140,000		Leenen et al., 1985
	Pyramidal tract (brainstem)		133,000	12	Leenen et al., 1982
	Pyramidal tract (cervical)	396,200	35,000	13	Leenen et al., 1985
Cat	Pyramidal tract	20,768	47,645	12	Biedenbach et al., 1986
	Pyramidal tract	10,696 and 15,166	20,750–63,290	8 and 15	Thomas et al., 1984
	Dorsal funiculus, sacral 2		8,488 ± 845	29	Chung et al., 1985
	Dorsal funiculus, sacral 2		19,559 ± 1,465	36	Chung et al., 1985
	Lateral funiculus, sacral 2		126,763 ± 29,858	44	Chung et al., 1985
	Ventral funiculus, sacral 2		32,182 ± 6,587	30	Chung et al., 1985
	WM sacral 2			45	Chung et al., 1985
	Tract of Lissauer, sacral 1 and sacral 3		49,500	80	Chung et al., 1979

*All those studies have been conducted with electron microscopy*.

In rats, about 12% of the fibers in the CST seem to be unmyelinated (Brösamle and Schwab, 2000), as opposed to about 60% in the pyramidal tract (Ralston et al., 1987). In terms of numbers, the cervical portion of the CST of the rat presents only 35,000 fibers against 140,000 for the pyramidal tract at the medullary level (Leenen et al., 1982, 1985). This decrease is mainly due to corticobulbar projections terminating in the brainstem.

In cats, a similar proportion of umyelinated fibers can be found as 12% of the pyramidal tract is unmyelinated at the medullary level. However, in the tract of Lissaeur, this proportion reaches around 80% at mid-thoracic and lumbosacral levels. At sacral levels, Chung et al. (1985) shows that 45% of the SC WM is unmyelinated in the cat, a surprisingly high level of myelination. It has to be noted that a high concentration of unmyelinated fibers can be found at the tract of Lissaeur and in the dorsal funiculi, and that propriospinal fibers could also participate in this high unmyelinated count (Chung et al., 1979; Thomas et al., 1984; Biedenbach et al., 1986).

As opposed to those species, monkeys appear to present very few unmyelinated axons (<1%) in the pyramidal tract (Ralston et al., 1987).

As can be expected, fibers count drops considerably from the medullary pyramids to the cervical levels of the CST: (Leenen et al., 1982, 1985). At a given spinal level, the type of tract also plays a role: for example in the cat, the lateral funiculus contains much more unmyelinated fibers (125,000) than the ventral or dorsal ones (30,000 and 20,000) (Chung et al., 1985).

#### Points of importance regarding unmyelinated axons characteristics

With the few numbers of studies on unmyelinated fibers morphometry, it is difficult to draw trends. Furthermore, studies have been conducted in the pyramids in the medulla oblongata, inducing a possible bias with the presence of corticobulbar fibers in the pyramidal tracts. Secondly, another bias could arise with a possible confusion between unmyelinated fibers and glial processes therefore overestimating unmyelinated fibers counts (Ralston et al., 1987). Also note that dendrites can also be found in the WM (Ralston et al., 1987). Confusion of structure however remains unlikely considering that studies were accomplished with transmit electron microscopy (TEM), providing high resolution and hence easy identification of cell structures.

The available information would perhaps suggest a decrease in unmyelinated fibers in the CST along the phylogenetic tree. This reduction could be correlated with the use of distal muscles to accomplish fine tasks (Ralston et al., 1987), or as a compensatory mechanism for the longer distances. Another possibility, at lower level than the medulla, would be the presence of propriospinal fibers running along several levels of the SC (Chung et al., 1985). Lastly, those unmyelinated fibers may also be collaterals from myelinated projections and with the increase of direct corticomotor connections, the number of those collaterals may decrease in primates (Schieber, 2007). Nevertheless, further additional studies are required to confirm the variation in unmyelinated fibers and their identification.

### General comments on previous literature

#### Major interest in the pyramidal tract and corticospinal tract

Most studies on neuroanatomy have been conducted on the pyramidal tract. It is the most studied pathway in the CNS, although some articles focused more on the CST (Leenen et al., 1982; Brösamle and Schwab, 2000). This tract is heavily involved in the motor control of the body, even more so in primates and humans (Thomas et al., 1984; Brodal, 2010; Firmin et al., 2014). In addition to its important physiological role, the pyramidal tract is also appealing since it can be rather easily identified at the level of the medulla (Thomas et al., 1984). As for the other tracts, very little information is available: the fasciculus gracilis in the SC dorsal column is reported to contain predominantly small diameter axons, whereas other tracts such as the corticospinal in the lateral columns and the ventral columns contain a mixture of small and large diameter axons (Johansen-Berg and Behrens, 2013).

#### Difficulty of assessing the type of pathway

**Figure 2:**
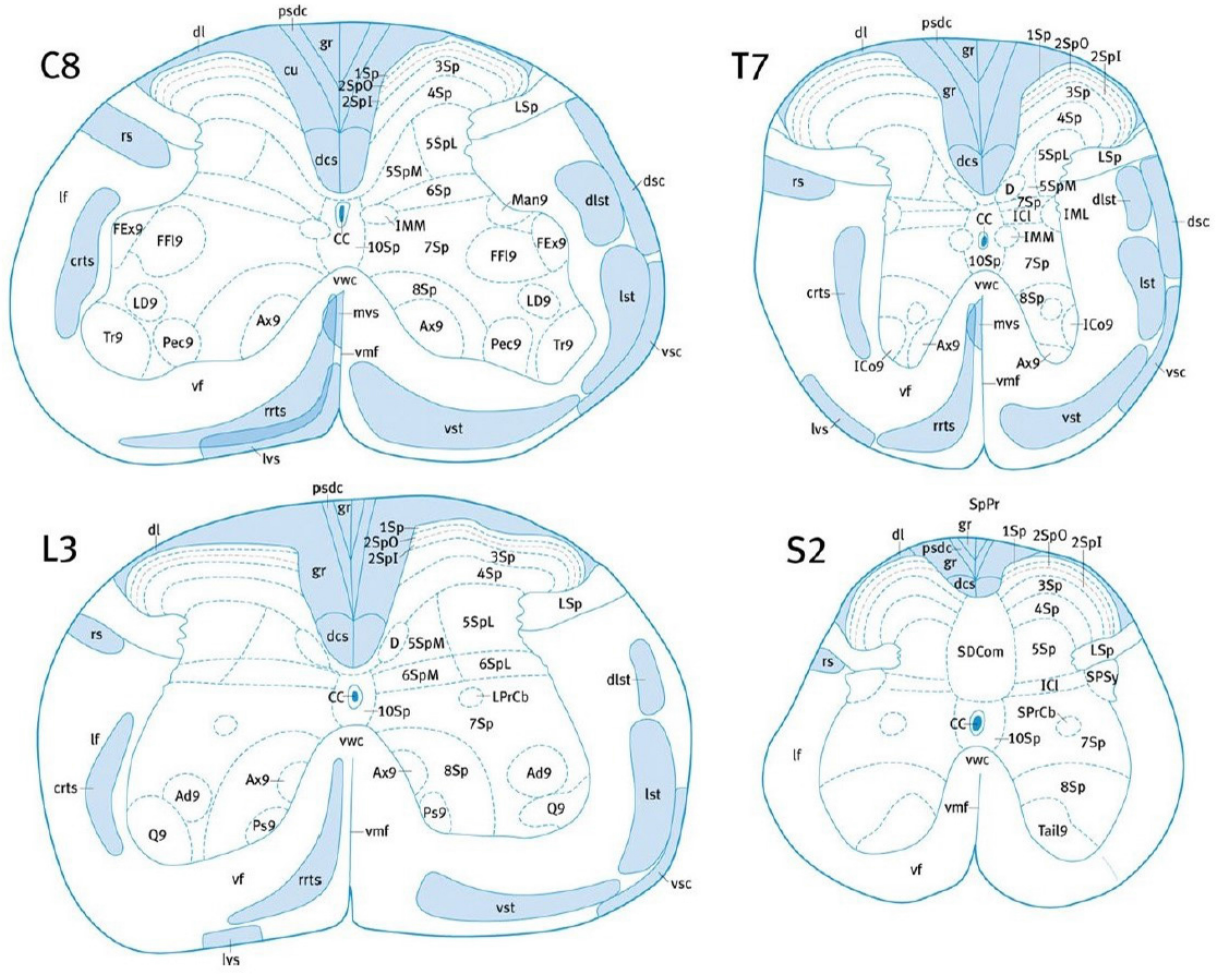
Localization of the major white matter tracts of the mouse SC, at C8, T7, L3, and S2 levels. Adapted with permission from (Watson and Harrison, 2012). 1Sp, lamina 1; 2Spl, lamina 2 inner part; 2SpO, lamina 2 outer part; 3Sp, lamina 3; 7Sp, lamina 7; CC, central canal; crts, caudal reticulospinal tract; cu, cuneate fasciculus; dcs, dorsal corticospinal tract; dsc, dorsal spinocerebellar tract; dl, dorsolateral fasciculus; dlst, dorsolateral spinothalamic tract; gr, gracile fasciculus; IML, intermediolateral column; lst, lateral spinothalamic tract; LSp, lateral spinal nucleus; lvs, lateral vestibulospinal tract; mvs, medial vestibulospinal tract; pdsc, postsynaptic dorsal column pathway; rrts, rostral reticulospinal tract; rs, rubrospinal tract; SPSy, sacral parasympathic nucleus; vsc, ventral spinocerebellar tract; vst, ventral spinothalamic tract.

Many studies rely on subjective approximate location of tracts based on previous atlas work and on the researcher's judgment, which necessarily adds uncertainty and bias. The variability in the location of the CST amongst species requires the use of an atlas established precisely for each particular species, at each spinal level (Leenen et al., 1985; Watson et al., 2009; Meurant, 2011; Watson and Harrison, 2012). Nevertheless, tract location and size could also be subject to inter-individual variability (Watson et al., 2009; Meurant, 2011). In addition, some tracts may overlap, such as the lateral vestibulospinal tract, the ventral spinothalamic tract and the rostral reticulospinal tract in the mouse (Figure 2). Lastly, the possible presence of ascending fibers in some tracts could also influence the analysis (Leenen et al., 1982, 1985; Thomas et al., 1984).

To be certain about the pathway observed, studies would require tracing methods in order to obtain a precise identification (e.g., di-I, dextran amines, Fluoro-Gold, PHA-L) (Köbbert et al., 2000; Reiner et al., 2000; Sparks et al., 2000; Vercelli et al., 2000; Altman and Bayer, 2001). The main drawback for tracing methods is the short penetration obtained in general (Sparks et al., 2000). Furthermore, with this technique tagging precisely the complete tract and preparing the tissue for observation can be complicated (e.g., washing out of diI during dehydration) (Köbbert et al., 2000; Reiner et al., 2000; Vercelli et al., 2000).

Some articles such as the one by Harding and Towe (1985) used a simpler method: they identified the pyramidal tract as the region presenting a high density of fine fibers, with only a few coarser fibers scattered throughout. To identify the border with the medial lemniscus, a large bundle of heavily myelinated ascending axons, the latter was characterized as composed of coarser fibers but they mentioned the difficulty of this technique in establishing a clear boarder. The efficacy of this protocol is indeed debatable, since the medial lemniscus covers the dorsal part of the pyramids (van Crevel and Verhaart, 1963; Biedenbach et al., 1986). For some tracts this technique could be applicable such as in rodents where the dorsal CST is rather well defined, bordered by the dorsal horns and presenting a clear difference in densities and axon diameter to the cuneatus, another tract also quite easy to identify (Hanaway et al., 1998; Standring, 2008). Yet, this technique is not applicable to all SC tracts (such as the rubrospinal pathway or the lateral corticospinal pathway) as it will be too imprecise (Standring, 2008). Other papers do not even mention their identification protocol, preventing readers to evaluate the biases that could exist.

A way to avoid the problem of assessing the pathway would be to do an analysis on a rougher compartmentalization of the SC, comparing or singling out wider regions of the SC. One could separate the SC in three blocs: dorsal, lateral and ventral in a similar fashion to Chung et al. (1985). The main drawback of this approach is that it does not bring precise information on the composition and exact morphology of each tract. On the other hand, this type of study would be very interesting for validation purposes, for example for new quantitative MRI studies.

Literature contains few microstructural studies on the WM of the SC and rather focuses on the pyramidal tract in the brainstem. Data on axonal morphometry in SC is therefore scarce, and the predominant interest toward the corticospinal tract is at the expense of other tracts. There is also very little data regarding myelin sheath thickness in the literature. Out of the microstructural studies published, a clear problem appears: the lack of homogeneity in the findings, whether across studies or in the same study. The next section addresses the potential sources of variability.

## Sources of variability

Different factors could explain the high intra- and inter-study variability described in the previous section. Taking them into consideration is important to be able to establish trends in morphometry. It is also important for injured SC morphometry as it could obscure effects of the pathologies. For example, body weight has been shown to influence the number of fibers in the pyramids within species, and could participate in high-inter-individual variability of the CST (van Crevel and Verhaart, 1963; Towe and Luschei, 2013).

### Age

Several studies about the effect of aging in different nervous tissue have been published and are summarized in Table 5.

**Table 5:** Evolution of morphometric data obtained from nervous tissue according to age in different species.

**Species/tissue**	**Number of fibers**	**Density**	**Mean axonal diameter**	**Myelin sheath**	***g*-ratio**	**References**
Human spinal cord L1	⇓		⇓			Zhou et al., 1997
Human fasciculus gracilis C3	⇓					Ohnishi et al., 1976
Human corticospinal tract		⇓ (small fibers)				Terao et al., 1994
Human corticospinal tract		⇓				Nakanishi et al., 2004
Human sural nerve	⇓ but ⇑ for small axons	⇓		Constant until ≅ 60		Jacobs and Love, 1985
Human optic nerve	Constant		Preferential large fiber loss			Repka and Quigley, 1989
Human superior laryngeal nerve	Constant (myelinated fibers)		No significant difference			Tiago et al., 2007
Human superior laryngeal nerve	⇓ (myelinated fibers)	Constant	⇓ of 1–2 μm fibers and 0–0.5 μm axons			Mortelliti et al., 1990
Human recurrent laryngeal nerve	⇓ (myelinated fibers)		⇓ of 1–3 μm axons			Tiago et al., 2007
Monkey optic nerve		⇓	Constant			Morrison et al., 1990
Monkey optic nerve	Constant		⇑			Fortune et al., 2014
Mice olfactory nerve		Constant	⇓			Watanabe et al., 2007
Rat hypoglossal nerve (male)	Constant (myelinated)		⇓	⇓	⇓	Soltanpour et al., 2012
Rat sural nerve	Constant for myelinated	⇓ (myelinated)	⇑ myelinated and axon diameter	Myelin sheath area ⇑	Constant	Jeronimo et al., 2005
Rat brain			⇑	⇑for F ⇓for M		Yang et al., 2008

*F, female; M, male*.

In the human SC, fiber counts and mean axonal diameter (Ohnishi et al., 1976; Zhou et al., 1997) as well as fiber density (Terao et al., 1994; Nakanishi et al., 2004) are believed to decrease with age. Whether this decrease in density is preferential to small vs. large fibers is debated (Terao et al., 1994; Nakanishi et al., 2004). With respect to human nerves, results are diverse and often contradictory. For the human superior laryngeal nerve, axonal counts have been reported as decreasing with age (Jacobs and Love, 1985; Mortelliti et al., 1990) or remaining constant (Tiago et al., 2007). The same trends were observed in animals, with decrease of fiber density with age. However, large discrepancies were found, for examples in monkeys (Morrison et al., 1990; Fortune et al., 2014), and rats (Jeronimo et al., 2005; Watanabe et al., 2007; Soltanpour et al., 2012). The tissue (e.g., PNS vs. CNS) and the species of origin may influence the evolution of morphometric parameters in regard to aging.

The effect of aging in the myelin sheath is poorly studied. One comment by Jacobs et al. suggested that thin or medium sized axons showed a disproportionately thick myelin sheath in older humans (>60 years old), consisting in loss of the expected relationship between axonal diameter and myelin sheath thickness (Jacobs and Love, 1985).

### Gender

Table 6 describes the effect of gender on axon morphology. Of particular interest, the study of Yang et al. (2008) showed that in young mice, males presented a higher total volume of myelinated fibers and myelin sheath than females did. A significant decrease in those parameters in male with aging was reported, whereas they increased with age in females. No sex difference for mean myelinated fiber diameter was found. They hypothesized that the increase in mean fiber diameter in males could be caused by the loss of the smaller fibers. In females, the increase in myelin sheath thickness could be explained by split of myelin lamellae, because of a shorter life span of oligodendrocytes. In a similar fashion, a group showed the importance of age on the SC but later (Zhou et al., 2000) refined their findings by specifying that the age-related reduction in axon diameter was seen in males only (Zhou et al., 1997, 2000). In the mice SC (cervical level), Cerghet et al. (2006) showed smaller levels of oligodendrocytes in females than in males as well as reduced myelin basic protein, hinting at a lesser degree of myelination in females (Cerghet et al., 2006). Interestingly, other studies did not find a significant effect of gender on axon morphometry (van Crevel and Verhaart, 1963).

**Table 6:** Evolution of morphometric data obtained from nervous tissue according to sex in different species.

**Species/tissue**	**Number of fibers**	**Density**	**Myelin sheath**	**Mean axonal diameter**	**g-ratio**	**References**
Human laryngeal nerve				M > F	M > F	De Campos et al., 2014
Rat phrenic nerve	Myelinated: F > M	Myelinated: F > M	Similar		Similar	Rodrigues et al., 2011
Rat brain			Young: M > F Old: F > M	Similar (myelinated)		Yang et al., 2008
Mice spinal cord cervical ventral funiculus			OLG: M > F MBP levels: M > F			Cerghet et al., 2006

*F, female; M, male; OLG, oligodendrocytes*.

Sexual dimorphism has already been established in the brain (particularly in the hippocampus) so even if to this day this issue has not been much addressed in the SC, it is likely that sexual dimorphism might be present in there too (Bear et al., 2007). For example, the sacral level in the SC differs from males to females since the former have supplementary motoneurons in the gray matter in order to control bulbocavernous muscles involved in ejaculation (Breedlove and Arnold, 1980; Forger and Breedlove, 1986; Wagner and Clemens, 1991; Bear et al., 2007). In addition, it seems that aging and sex would be interdependent factors for morphometry.

### Vertebral and spinal levels

The number of fibers in the WM will necessarily vary according to the rostro-caudal location in the SC, with descending fibers reaching their targets and ascending fibers entering progressively (Standring, 2008). Ohnishi et al. (1976) have indeed reported a decrease in the number of myelinated fibers of the fasciculus gracilis in human between C3 and T5 (respectively 25,267 and 23,069 per mm^2^). In general, more studies covered cervical segments than thoracic and lumbar ones.

The variation of axon diameter regarding the vertebral level is not clear so far: Leenen et al. (1985) reported no difference in the rat pyramidal tract, whereas differences were reported in the human lateral CST (Terao et al., 1994) and in the human fasciculus gracilis (Ohnishi et al., 1976).

### Statistical power

Intra-species variability seems to be high, as noted by Verhaart (1947), van Crevel and Verhaart (1963), Jacobs and Love (1985), and Morrison et al. (1990). It is informative to look at the number of animals used in each article. For instance, Harding's results (Harding and Towe, 1985) are based on a total of two rats; Biendenbach's (Biedenbach et al., 1986) on three cats; Firmin et al. (2014) used two monkeys for the neuroanatomical part of his article. Leenen's study (Leenen et al., 1985) in rats was realized on five animals, similar to Ralston's study (Ralston et al., 1987) studying six monkeys. In addition to the relatively low number of animals per study, because most studies used TEM, in general only 1–6% of the area of the tract is considered in the analysis. Partial sampling can introduce variability and error in addition to not being representative (Fortune et al., 2014).

### Protocols

Table 7 exemplifies some protocols used for characterizing axon morphology. The fairly large variability of protocols can partly explain the variability observed in the reported results across studies. Indeed, the choice of protocol can have several impacts on shrinkage, distortions and other artifacts. Another typical example that often occurs when imaging deep tissue, is that imperfect fixation of the myelin sheath with osmium (due to poor penetration) can induce lamellae separation (Harding and Towe, 1985).

**Table 7:** Histological protocols for ultrastructural observation of nervous tissue in literature.

**Species**	**Perfusion**	**Immersion**	**Osmification**	**Additional staining**	**References**
Monkey	2% Glutaraldehyde – 2% PFA				Ralston et al., 1987
	1% glutaraldehyde – 1% PFA	1% glutaraldehyde – 1% PFA	Osmium tetroxide 1% (1.5 h)	1% toluidine blue (OM) 1% uranyl acetate and 1% lead acetate (EM)	Firmin et al., 2014
Cat	1.5% glutaraldehyde – 1% PFA – 0.1% picric acid	1.5% glutaraldehyde – 1% PFA – 0.1% picric acid	Osmium tetroxide 1–1.5% potassium ferricyanide	1% uranyl acetate and 0.1% lead citrate (EM)	Biedenbach et al., 1986
Rat	0.5% PFA – 1.5% glutaraldehyde – 0.1% picric acid	0.5% PFA – 1.5% glutaraldehyde – 0.1% picric acid	Osmium tetroxide 2–0.15% potassium ferrocyanide (EM)	5% toluidine blue (OM) Uranyl acetate and lead citrate (EM)	Leenen et al., 1985
	0.5% glutaraldehyde – 4% PFA	0.5% glutaraldehyde – 4% PFA (2nd animal)	Osmium tetroxide 2% (1st animal) Osmium tetroxide 4–1.5% potassium ferrocyanide (2nd animal)	Methylene blue-azure II (1st animal) Methylene blue-azure II (OM) or 4% uranyl acetate and 11.6% lead acetate (EM) (2nd animal)	Harding and Towe, 1985
Human	10% formalin (3.7–4.0% formaldehyde) through the femoral artery	10% formalin (1 week) 5% potassium dichromate and 5% potassium chromate (2 weeks)		luxol fast blue-periodic acid Schiff-hematoxylin (LPH)	Souma et al., 2009

Even with an optimal protocol, issues remain such as microscope resolution. The theoretical resolution with light microscopy is ~300–350 and ~200–250 nm with numerical aperture of 1 (in air) and 1.5 (in oil), respectively (Bart, 2006; Madou, 2011; Nadeau, 2012; Bradshaw and Stahl, 2015), although in practice a resolution below 1 μm is hard to obtain (Madou, 2011). As a result, approximately 20% of fibers are not detected when imaged with light microscopy (Arbuthnott et al., 1980; Biedenbach et al., 1986; Ralston et al., 1987; Firmin et al., 2014). To reduce the mismatch between neuroanatomical and electrophysiological measures of fibers diameter, Firmin et al. (2014) used electron microscopy to show that many more small axons (with a diameter of 0.5 μm and less) were detected in comparison with previous studies that used light microscopy (Arbuthnott et al., 1980; Harding and Towe, 1985; Hildebrand et al., 1993; Firmin et al., 2014). The difficulty in detecting small fibers (less than 1 μm) comes with the challenge of accurately obtaining morphometry information such as diameter and myelin sheath thickness (Biedenbach et al., 1986; Firmin et al., 2014).

Lastly, most studies have employed a manual fiber identification and manual selection of the profile of interest. This procedure may induce a bias against small or ill-defined fibers (Biedenbach et al., 1986). Furthermore, the time needed to do this manual identification produces exhaustion-related bias and more importantly limits the area studied (More et al., 2011; Reynaud et al., 2012; Isaacs et al., 2014). For instance, Firmin et al. only sampled 0.015 mm^2^ of the tract in EM when the complete acquisition window was of 3.782 mm^2^ (Firmin et al., 2014).

There is a need for a thorough investigation of SC morphometry with high statistical power. To achieve this goal, analysis of the complete section of the SC (as opposed to samples) from several animals is necessary to provide a good overview of the inter-individual variability within species when all factors are accounted for (sex, weight, etc.). Due to the large area of the SC however (~1 cm^2^ in human) and the small size of fibers (~1 μm), a fast imaging technique at high resolution is necessary. To analyze these images, automatic and accurate segmentation softwares are needed.

## Suggested protocol for preparing white matter tissue

As previously discussed, many factors can affect the quality of sample preparations such as the fixation method, fixative concentration, duration of fixation, temperature and pH of fixation, solution osmolarity, staining, buffer, dehydration, and embedding. Suggestions for optimal protocol are listed hereafter, which are based on existing literature and on the authors' research experience.

### Fixation method

The tissue can be fixed in two ways. The first method, called immersion fixation, consists of cutting the tissue into small pieces which are then submerged into fixatives until the tissue is set in place. The cutting of the sample into small pieces should be done with a razor blade by one quick slashing motion instead of several back-and-forth movements in order to avoid any tissue damage. The second method, called perfusion fixation, involves delivering the fixatives through the systemic circulatory system. Perfusion flowrate should be adjusted according to the blood pressure: a pressure too high could lead to vessels exploding, while a low pressure could have a detrimental effect on fixation speed.

It is generally accepted that vascular perfusion fixation is a necessary step for tissues that undergo rapid lysis after removal and for providing well-preserved axons and myelin (Palay et al., 1962; Williams and Jew, 1975; Langford and Coggeshall, 1980; Lamberts and Goldsmith, 1985; Chang and Slikker, 1995; Fix and Garman, 2000). Thus, if perfusion cannot be done (e.g., human tissue), the tissue needs to be immersed in the fixative solution as soon as possible after death in order to minimize tissue damage.

### Fixative concentration

A mixture of glutaraldehyde (Ga) and paraformaldehyde (PFA) is recommended for ultrastructural studies as it combines the high cross-linking ability of Ga and the fast action of the PFA (Williams and Jew, 1975; Lamberts and Goldsmith, 1985; Chang and Slikker, 1995; Fix and Garman, 2000; Brancroft, 2008; Hayat, 2012). The original paper by Karnovsky (1965) recommends a mixture of 5% glutaraldehyde and 4% formaldehyde, which is a higher concentration than what people typically use (Hayat, 2012). Other empirically-determined mixtures that are widely used for both structural and immunocytochemical studies consist of a modified Karnovsky solution by using it in half-strength formulation; 2% PFA and 2.5% Ga in 0.1 M Sodium Cacodylate (or phosphate buffer with a pH of 7.4) (Karnovsky, 1965; Dykstra and Reuss, 2003). The optimal concentration of Ga and PFA is difficult as the chemical interactions of these two fixatives with each other and with the tissue are still not completely understood (Glauert and Lewis, 2014). A relatively wide range of Ga concentrations (about 1.5–4%) has been used in animal tissue fixation (Hayat, 2012). In general, a low concentration of fixative requires a longer duration of fixation, but long Ga fixation (over a week) can cause tissue shrinkage (Hayat, 2012). High concentrations (>4%) are also unsuitable since they destroy the ultrastructure (Bozzola and Russel, 1992). The presence of Ga affects the process of protein cross-linking and the osmolarity. High Ga concentrations (30% and more) can lead to severe shrinkage whereas low Ga concentration (0.5% and below) can cause severe cell components extraction if osmolarity is not controlled (Dykstra and Reuss, 2003; Hayat, 2012). Such extreme osmolarity of the fixative leads to myelin shrinkage, swelling or vacuolation.

Note that Ga should not be used with coherent anti-stokes Raman spectroscopy (CARS) imaging because it discards the anti-stokes effect (Bélanger et al., 2009). In this case, Ga can simply be discarded or replaced with acrolein.

### Duration of fixation

The penetration of fixatives into the tissues is described by Fick's second law (Yong-Hing et al., 2005; Thavarajah et al., 2012): $\frac{\partial c_{i}}{\partial t}=D\frac{\partial c_{i}}{\partial x}$ where *i* is fixative considered, *c*_*i*_ the fixative concentration, *x* is the axis of penetration, *t* the time of fixative penetration and *D* the diffusion coefficient of the fixative. Assuming that the volume of a fixative is large enough and the nominal concentration c_0_ of the solution is unchanged at the boundary of the tissue, the fixative concentration is:

$$\begin{array}{l} c_{i}\left(x,t\right)=c_{0}\left(1-\mathrm{erf}\left(\frac{x}{2\sqrt{Dt}}\right)\right)\approx c_{0}\left(1-\frac{x}{\sqrt{Dt}}\right) \end{array}$$

Let's define *d*, the depth of penetration at which 50% of the nominal concentration (*c*_*i*_(*d, t*) = 1/2*c*_0_) is obtained. We can write d ≈ 1/2$\sqrt{Dt}$.

The diffusion coefficient D is influenced by temperature (T), fluid viscosity (η), the size of the chemical compounds (r) according to the Stokes-Einstein law for solutions with low Reynolds numbers: $D=\frac{k_{B}T}{6\pi \eta r}\;$where *k*_*B*_ is Boltzmann's constant (Mehrer and Stolwijk, 2009). Thus, high temperatures, low fixative solution viscosity and small chemical compounds will increase the rate of diffusion.

The diffusion values for osmium tetroxide (OsO_4_), Ga and formaldehyde are respectively *D* = 0.2, 0.34 and 2.0 10^−9^ m^2^/s (Johannessen, 1977). Thus, using $d\approx 1/2\sqrt{Dt}$, at room temperature, Ga and PFA penetrate the tissue by 1 and 3 mm respectively after 4 h, or by 3 and 7 mm after 24 h.

Regarding Osmium penetration, the reaction with unsaturated lipids drastically reduces the permeability of the membranes and thus the diffusion within the tissue. Osmium staining is thus strongly reduced after 200 μm. Strategies have been developed to improve Osmium penetration, by reducing temperature during the osmification process (osmium reaction is more greatly reduced than diffusivity) or/and by using a reducing agent to improve contrast (Mikula et al., 2012; Hua et al., 2015)

### Temperature and PH

At low temperatures, the penetration rate is slowed down, as well as the reaction of the fixatives (which affect the permeability of the tissue membranes). At high temperatures autolytic changes occur more rapidly (Hunter et al., 1993) which cause the formation of fixation artifacts (e.g., cell shrinkage, cell volume and shape changes) (Hayat, 2012). Some investigators demonstrated that formaldehyde fixation at room temperature induces poor preservation (Cross et al., 1990). However, depending on the type of tissue, Ga can be used at temperatures ranging 0–25°C with little morphological differences in the ultrastructure (Hayat, 2012). Regarding perfusion, cold temperatures affect some metabolic processes and can induce loss of these structures (Hayat, 2012). In vascular perfusion, using a Ga solution below body temperature may cause vasoconstriction (Fix and Garman, 2000).

When cells die during the fixation process, the lysosomes release their contents and make the pH of the tissue acidic. The optimal pH is close to that of the tissue and has to be maintained during the entirety of the tissue preparation process. Indeed, a change in the tissue pH could severely alter its ultrastructure. Since the average pH of most animal cells is 7.4 it is recommended to adjust the pH of the fixative solution to the physiological range of 7.2–7.5, especially when using Ga or osmium tetroxide (OsO_4_) (Peters, 1970).

### Osmolarity

The osmolarity of a fixative solution has a direct effect on the myelin morphology. Axon size and shape may be affected by the buffer or the fixative solution's osmolarity. Osmolarity depends on the type of buffer and the concentration of the fixatives (Schultz and Karlsson, 2006). At low osmolarity (≤320 mOsmol), the fixation is very poor and causes cell swelling. The osmolarity of the fixative solution should be slightly hypertonic (400–600 mOsmol) to prevent blood vessels from bursting during perfusion (Fix and Garman, 2000; Schultz and Karlsson, 2006). Higher osmolarity (>1,000 mOsmol) could induce considerable tissue shrinkage and widening of the extracellular space (Ohnishi et al., 1976). As the resistance in blood vessels increases during perfusion, the fixative flow stops rapidly through the specimen, resulting in a very poor fixation (Ohnishi et al., 1976; Schultz and Karlsson, 2006). Electrolytes, especially CaCl_2_, when added to isotonic fixative solutions can overcome the issues of osmolarity and decrease fixation artifacts such as swelling and shrinkage (Hayat, 2012). To increase the osmolarity, saccharose or tannic acid can be added to the fixative (Hayat, 2012).

### Osmification

Osmium tetroxide (OsO_4_) has two interesting effects: it fixes the lipids and it stain the membranes for optical or electron microscopy. Without osmium, lipids are dissolved in the dehydration process (Robertson, 1957; Langford and Coggeshall, 1980; Dykstra and Reuss, 2003; Bruce-Gregorios, 2006; Fujimoto et al., 2012; Glauert and Lewis, 2014). In electron microscopy osmium improves the back-scattered electron density thanks to its large atomic number (Z = 76) and the conductivity of the sample's surface (Bozzola and Russel, 1992). As discussed earlier, osmium has a short penetration depth, and it is thus recommended to use a small sample size in one dimension or to image a slice close to the surface (<200 μm). Note that other electron-dense stains exist, such as uranium salts, potassium permanganate and ruthenium tetroxide (Hayat, 2012).

### Dehydration

After being post-fixed in osmium tetroxide, the specimen needs to be dehydrated at room temperature. The purpose of dehydration is to remove water from the tissue so that the embedding media can infiltrate the tissue. Ethanol (mixed with propylene oxide) or acetone solvent are commonly used. Some electron microscopy textbooks suggest that ethanol with propylene oxide present better infiltration than acetone and would be recommended for large samples (Bozzola and Russel, 1992; Glauert and Lewis, 2014). However, propylene oxide is more toxic than ethanol and acetone appears to cause less shrinkage than ethanol (Dykstra and Reuss, 2003; Rana, 2008).

### Embedding

As acrylic resin is harder than paraffin, the use of this embedding media allows for cutting thinner sections than with paraffin (0.1–2 vs. 3–6 μm), in turn allowing greater resolution for optical microscopy (Sheehan and Hrapchak, 1980; Bancroft and Gamble, 2002; Lowe and Anderson, 2014).

In order to cut the hard acrylic resin, diamond or tungsten-carbide knifes can be used. Diamond knives are used to cut ultra-thin sections for TEM (<100 nm), but they are usually only a couple of millimeters long and can only cut samples of that size range. Tungsten-carbide blades can cut larger samples however produces thicker slices (~1 μm).

The choice of epoxy resin is determined by its hardness, its viscosity (and thus its penetration), its cutting behavior (very homogeneous resins are easier to cut), and its resistance under the electron beam. “Embed 812” is a standard epoxy resin that satisfies the above-mentioned criteria.

## Novel techniques and perspectives

Methods for preparing tissues and analyzing their ultrastructure have not fundamentally changed since they were first developed in the 1960s. However, recent techniques have been introduced that can improve the current state-of-the-art of mapping axon morphometric in the SC (Mikula and Denk, 2015). This section lists some of the new technologies that feature large-scale microscopy, endogenous contrast to myelin, and analysis software for axon and myelin segmentation.

### Coherent anti-stokes Raman scattering (CARS)

Currently, the observation of axons and myelin sheath relies on the conventional stains mentioned above. However, the preservation of myelin ultrastructure is strongly sensitive to fixation artifacts. Therefore, new myelin imaging techniques have been developed during the last decades that can be used without fixing the tissue or using immunostaining with myelin basic proteins (MBP) and other agents that can identify the ultrastructure *in situ* (fluorescence) (Wang et al., 2010; Bajaj et al., 2013).

**Figure 3:**
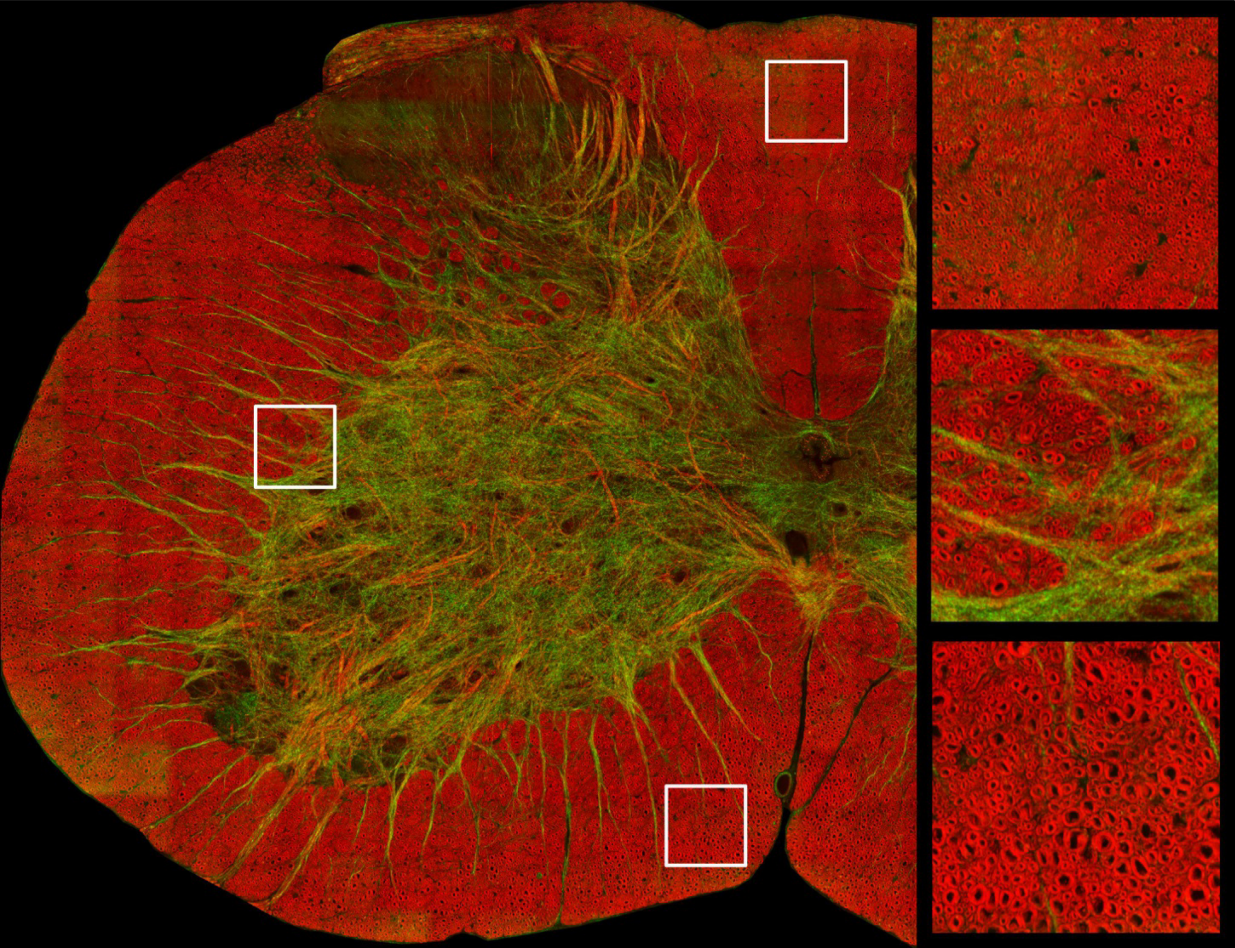
Image of a cross-section of a mouse spinal cord based on coherent anti-stokes Raman spectroscopy, which is tuned to exhibit signal from myelin sheath. Axial axons appear in red, while lateral fibers and some vessels appear in green. Zoomed panels focus on the dorsal **(top right)** and ventral white matter **(bottom right)** and in the ascending reticulospinal/fasciculus proprius region **(middle right)**. Courtesy of Erik Bélanger, Sophie Laffray and Daniel Côté.

Coherent Anti-Stokes Raman Scattering (CARS) is a recent imaging technique that has proven useful for imaging myelin (Wang et al., 2005; Bélanger et al., 2009). This modality is sensitive to the biochemical nature of tissues. When the frequency difference between two laser beams targeting a sample is resonant with the Raman vibrations of lipid (CH2 at 2,850 cm^−1^), the lipids that make the myelin membrane emit light. Axial and lateral CARS resolution are respectively about 0.70 and 0.28 μm (Wang et al., 2005). As CARS relies on the endogenous chemical contrast provided by lipids in the myelin sheath it abolishes the need for staining and dehydration/embedding (the critical step for myelin damage), therefore allowing for more realistic measures of g-ratio and myelin sheath thickness (Wang et al., 2005; Bélanger et al., 2009; De Vito et al., 2015). Additionally, this technique can image myelin *in vivo*, allowing researchers to follow demyelination in animal models of MS or nerve crush injury (Henry et al., 2009; Bélanger et al., 2011; Imitola et al., 2011). An example of a CARS image is shown in Figure 3.

### Optical coherence microscopy (OCM)

Optical Coherence microscopy uses the backscattered light due to refractive index variations in the tissue as a source of endogenous contrast. Myelin produces a high intrinsic backscattering signal due to the high refractive index of the lipid-rich myelin sheath. Just like CARS, OCM performs virtual sectioning by selecting a slice (through interferometry) at a depth of up to one hundred microns. Preventing the sectioning step prevents tissue damage and allows *in vivo* imaging (Ben Arous et al., 2011). Axial and lateral resolution can reach respectively 1.15 and 0.5 μm (Ben Arous et al., 2011). OCM provides not only quantitative measurements of myelin optical properties but also novel optical markers of cell viability and myelination (Srinivasan et al., 2012). The limited resolution, however, prevents fine measurement of axon morphometry.

### Third harmonic generation microscopy (THG)

Third harmonic generation (THG) microscopy is a coherent, nonlinear, dye-free imaging modality. THG can reach a micrometer resolution and has been used to monitor myelin loss and recovery in the mouse CNS (Farrar et al., 2011). Just like the two previous techniques, THG does not require any exogenous dye or fluorescent proteins and provides 3D structural images. It can be combined with other non-linear optic such as the two-photon excitation fluorescence (2PEF) and second harmonic generation (SHG). The resolution can be improved down to 5 nm if a super-resolution technique is used (Sandkuijl et al., 2013; Dashtabi et al., 2017).

### Immunostaining in combination with confocal microscopy

Immunostaining is an antibody-based method to detect a specific protein in the tissue. Immunostaining based on myelin basic proteins (MBP) in combination with confocal microscopy provides a sensitive and reliable method to observe the fine structure of myelin sheath (Xing et al., 2012).

Confocal microscopy has a pixel resolution around 0.3 μm (Conn, 2010). Using laser-scanning confocal microscope, axial and lateral resolution can reach 38 and 122 nm respectively (Potrusil et al., 2012), and can image a slice a couple of hundred microns deep in the tissue, allowing *in vivo* imaging. For electron microscopy, antibodies are labeled with gold particles that can be observed with TEM. However, immune-EM can be technically challenging, expensive and requires rigorous tissue fixation (Xing et al., 2012). Note that confocal microscopy does not necessarily require fluorescence labeling. Using multiple confocal lasers tuned at different wavelengths, Spectral Confocal Reflectance microscopy (SCoRe) can be specific to myelin, making the application of this technique easier on animals other than rodents (Schain et al., 2014).

### Slide scanner and stitching algorithms

Imaging large sections of a sample, such as a complete SC transection, has become possible through the use of a motorized stage combined with an algorithm that stitches together small field-of-view images acquired at high resolution. These large-scale images can then be observed and analyzed on a virtual slide viewer (Weinstein et al., 2009; Pantanowitz et al., 2011).

Alternatively, whole-slide scanners provide a high-speed standardized acquisition with predefined parameters, with the ability to image up to 200 slides in one night, in bright field or with fluorescence, at 20 or 40× (Weinstein et al., 2009; Nederlof et al., 2011; Pantanowitz et al., 2011; Ameisen et al., 2012; Laurent et al., 2013; Gallas et al., 2014). A recent example of the use of whole-slide scanners is the BigBrain: a whole human brain scanned at 20 μm resolution (Amunts et al., 2013).

### Automatic segmentation

Automated analysis has become important for large-scale morphometric studies, as manual identification of axons is long, tedious and subject to user bias (More et al., 2011). Several algorithms exist for axon and myelin segmentation (Romero et al., 2000; More et al., 2011; Bégin et al., 2014; Mesbah et al., 2016; Zaimi et al., 2016, 2017). Following segmentation of axon and myelin, metrics such as distribution of axon diameter, mean myelin thickness or myelin volume fraction can be more readily computed (Bégin et al., 2014).

**Figure 4:**
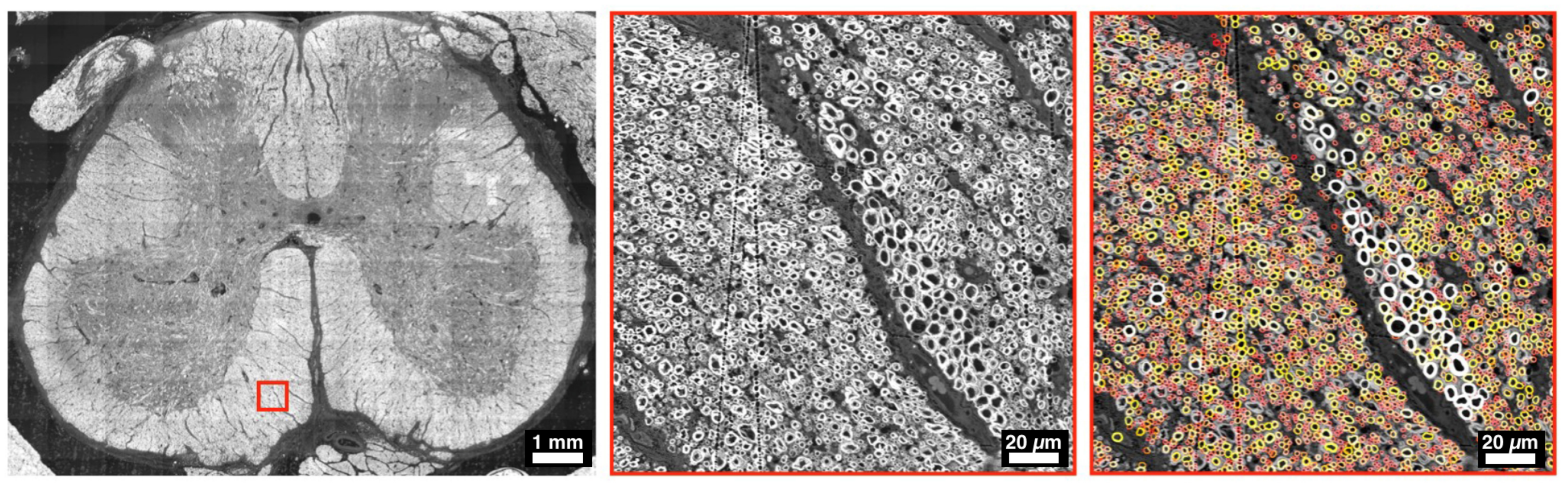
Example of a whole slice of a human SC at L5 level, imaged with electron microscopy. The middle panel focuses in the ventral region, and the right panel shows an overlay of myelin segmentation, color-coded for axon diameter (red: small, white/yellow: big). Segmentation was performed using AxonSeg (https://github.com/neuropoly/axonseg).

The use of axon segmentation software has shown encouraging results in terms of sensitivity and accuracy, especially when compared to manual identification (Reynaud et al., 2012; Isaacs et al., 2014). For example, Bégin et al. (2014) segmented 60% of the complete section of a mouse SC in 4 h, counting a total of 32,000 axons. More et al. (2011) have identified 84.3% of axons in a whole SEM slice of peripheral nerve (4 ms/axon) and were able to measure myelin sheath thickness and axon diameter. Figure 4 illustrates a semi-automatic segmentation on SC tissue.

A fully automatic approach with perfect accuracy is, however, difficult to achieve. For example, algorithms can sometimes fail to discriminate fibers that are too packed, or to distinguish between neuronal fibers and other tissue components such as blood vessels. Hence, manual intervention is often required (More et al., 2011; Bégin et al., 2014; Isaacs et al., 2014; Zaimi et al., 2016). Machine learning methods (e.g., deep learning algorithm), could potentially be trained to overcome these issues and avoid human intervention (Cireşan et al., 2013; Zaimi et al., 2017).

In addition to the segmentation issues, the metrics extracted are potentially affected by the quality of the image. The inherent point spread function of the imaging system, a bad focus, or an inhomogeneous contrast/illumination (e.g., due to inhomogeneous staining) can lead to over- or under-estimated morphological values (e.g., myelin thickness, myelin volume). An additional challenge is thus to use an imaging system that is highly stable on the entire slice.

## Concluding remarks

In view of the existing literature, complete cartography of the SC WM microstructure is still difficult to achieve. The conventional procedures for histology of the central nervous system are invasive (cutting, staining) and face a constant trade-off between resolution and field-of-view. Quantitative descriptions of WM axons are mostly concerned the corticospinal or the pyramidal tracts. Results, however, are highly variable across studies, which could be attributed to differences in protocols, terminology, precise location of the region of interest and user bias. With the recent development of high-resolution, dye-free, in depth, whole slide imaging systems, combined with stitching and fully-automated segmentation algorithms, reproducible and comprehensive measures of axon morphometry in the spinal cord can be obtained. These new techniques can help pave the way toward large-scale imaging and characterization of WM microstructure across species.

## Author contributions

BP conducted the literature review on axon and myelin and wrote the article. AS conducted the review on the suggested protocol and wrote the article. TD conducted the review on the novel perspectives and wrote the article. NS, SR, and JC-A wrote and reviewed the article.

### Conflict of interest statement

The authors declare that the research was conducted in the absence of any commercial or financial relationships that could be construed as a potential conflict of interest.
